# Frequency of provision of knowledge of performance on skill acquisition in older persons

**DOI:** 10.3389/fpsyg.2014.01454

**Published:** 2014-12-12

**Authors:** Marcelo E. S. Nunes, Marina G. T. X. Souza, Luciano Basso, Carlos B. M. Monteiro, Umberto C. Corrêa, Suely Santos

**Affiliations:** Laboratory of Motor Behaviour, School of Physical Education and Sport, University of Sao PauloSao Paulo, Brazil

**Keywords:** knowledge of performance, older persons, motor learning, feedback

## Abstract

The provision of feedback is a crucial factor for the evolution of the learner’s performance. It is known that the knowledge of performance has the function of guiding the learner’s attention to critical aspects of the movement pattern. The objective of this study was to examine the effect of frequency of knowledge of performance (KP) during the acquisition of the basketball free throw in older persons. Sixty active individuals (men and women) aged 60–69 years of age, divided into three experimental groups received KP in 100, 66, and 33% of their attempts during three practice sessions totaling 90 trials. The task was the basketball free throw. Volunteers were asked to conduct tests of immediate retention, 24 h retention, and 24 h transfer test, after the last practice session. During the acquisition phase, the volunteers received KP on the movement pattern on the previous attempt, which was obtained from a qualitative hierarchical checklist of the free throw (14 items). Sessions were recorded in order to confirm whether volunteers were able to score throughout sessions. ANOVA indicated that all individuals showed an improved performance in the retention and transfer tests. But the KP frequency of 66% was superior in both qualitative (movement pattern) and quantitative (score) measurements throughout the trials (*p* ≤ 0.05). In conclusion older persons seem to need an optimal KP frequency supply during the learning process.

## INTRODUCTION

With aging, the capacity to continue to learn new motor skills is of the utmost importance for maximizing quality of life, since older persons need to practice and learn new skills and to relearn motor skills that were practiced in the past; this is true whether the skills are part of a recreational activity, a training task or a rehabilitation task (Carnahan et al., 1996). Specifically, viewing a task as learnable, and performance as modifiable through practice, as opposed to something that reflects inherent and stable ability, can enhance the performance of the task (Wulf et al., 2012).

In motor learning studies using magnetic resonance imaging (MRI), some structural and functional changes were observed in the brains of older participants (Boyke et al., 2008; Bo et al., 2011; Lin et al., 2012; Turner and Spreng, 2012). In summary, these studies reveal that older persons continue to demonstrate brain plasticity over the course of the aging process.

On the other hand, one cannot ignore the fact that aging is accompanied by numerous changes, including a decrease in cognitive capacity (Williamson et al., 2009) and changes in the mechanisms of perception (Tia et al., 2010), in motor sequence learning (Bo et al., 2011; Nemeth and Janacsek, 2011), in the functioning of the sensory-motor system and in response speed (van de Laar et al., 2012).

Shephard (1997) has pointed out that the decline due to aging only means that the instructor/teacher must take a number of special steps at the moment of giving information in order for the skill to be acquired. This can be observed, for example, in the study by Carnahan et al. (1996), who showed that older persons who received extrinsic feedback, or knowledge of results (KR), after every five attempts were more consistent and precise in their performance the ones who received KR after each attempt. When the relationship between KR frequency and aging is considered (Behrman et al., 1992; Carnahan et al., 1996; Wishart and Lee, 1997; Gehring, 2008), the results do not complement each other, and nor do they clarify what is the ideal frequency of providing KR to the older persons.

The frequency of KR in the learning of motor skills has caused some authors to develop explanatory hypotheses for the phenomenon. The first hypothesis to be generated was the hypothesis of similarity or specificity, which proposes that the conditions during the acquisition phase, when similar to those during the retention phase, produce better results for learning (Henry, 1968). In this case, the groups that received feedback on all of the attempts during the acquisition phase would have their performance affected on undertaking the test of retention in the complete absence of KR. On the other hand, this condition of no provision of extrinsic feedback would favor groups that received reduced frequencies of KR during acquisition, since they would be exposed to similar conditions.

This hypothesis has been tested by some researchers in the field of motor learning (Winstein and Schmidt, 1990; Goodwin and Meeuwsen, 1995), and was rejected. As the results obtained in their studies did not support the hypothesis of specificity or similarity, Schmidt and Young (1991) sought to explain the superiority of reduced frequencies of KR provision to the detriment of higher frequencies of provision through the hypothesis of instability or maladaptive short-term corrections. According to this hypothesis, frequent feedback may lead the learner to an excessive instability during practice, causing frequent adaptations or modifications to performance, and consequently making it difficult to develop the capacity for stability on retention and on transfer. The provision of KR, attempt by attempt, leads to constant corrections by the learner and, thus, the individuals would fail to acquire consistency in the motor response. In other words, when KR is presented frequently, this leads the learner to make corrections even when the error was minimal, that is, to make unnecessary corrections, negatively impacting on learning. On the contrary, reduced frequencies of KR would lead to a consistency over the course of the attempts, providing a basis for the use of KR when this was presented (Chiviacowsky, 2005). A third hypothesis was proposed by Salmoni et al. (1984), named the guidance hypothesis. This hypothesis is based on the idea that the fact the learner receives KR on each attempt would lead to dependency on this information; inhibiting other important processing activities in the process of motor learning; inhibiting the capacity to detect and correct errors and causing a block in operations of memory recall which would threaten the development of the action plan.

In many learning or relearning environments, knowledge of performance (KP) is used instead of KR, when instructors want to direct the attention of the learner to the critical factors of the movement pattern or of the environmental context in which that pattern occurs. For Magill and Grodesky (2005), the provision of KP becomes particularly important when applied to learning in older person. This is because, in comparison with younger people, older persons pay attention to less information at a given moment, and remember this information for a shorter period of time.

Thus, bearing in mind the changes in information processing during aging, especially in terms of short-term memory, a relevant question is what frequency of KP positively influences the performance of older individuals during the learning process. On the one hand, a high frequency of KP can benefit individuals who present difficulties in maintaining the focus of their attention on the information given by the instructor. In contrast, a high frequency of KP may overload the capacity for information processing of them.

In summary, the association between these two factors, aging and KP, could be investigated by an analysis of the process through which an older individual learns a motor skill. For this reason, the objective of this work was to investigate the effect of the frequency of KP provision (given on 33, 66, and 100% of occasions) on the acquisition of a motor skill in older individuals.

## MATERIALS AND METHODS

### PARTICIPANTS

This study involved 60 volunteers, of both sexes, who were aged between 60 and 70 years old and were physically active; the study was approved by the Ethical Committee in Research of School of Physical Education and Sport, University of Sao Paulo. The exclusion criteria included having prior experience of the motor task employed in the study and achieving a score of less than 25 in the Mini Mental State Examination (MMSE; Folstein et al., 1975).

### TASK

The motor task executed by the participants was the basketball free throw; the participants had to stand behind a line at a distance of 2.30 m from the basket, and face the backboard. In the tests of retention, the distance was maintained, while, for the transfer test, the distance was 2.40 m, and the individuals were positioned at 45° to the left of the basket. During all phases of the experiment the basket was maintained at a height of 2.30 m from the ground.

### EQUIPMENT

The participants performed the throws using a *Penalty* brand junior ball; their target was a *Spalding* removable basketball backboard, with an adjustable height ring. During data collection, a notebook computer and a *Sony H50* video camera were used.

### MEASUREMENTS

The performance was analyzed through two measurements: one qualitative, involving the use of a checklist (**Table 1**) and being designed to assess the quality of the basketball throw; and one quantitative, being obtained from the result of the action mentioned above – each successful throw was awarded one point, and the remaining situations zero points.

**Table 1:** Checklist to assess the quality of the basketball throw (Nunes et al., 2012).

1	Right/Left throwing arm and corresponding foot ahead.
2	Bend the knees at the beginning of the movement.
3	Bring the ball closer to the chest.
4	Right/left elbow in line with the shoulder.
5	Hold the ball with the right hand from behind and the left hand at the side.
6	Palm of the hand looking up.
7	The support of the ball is only in the fingers.
8	Right/Left elbow pointing to the basket.
9	Look at the basket.
10	Extend your legs, trunk, and arms together.
11	At the end of the movement, the fingers should point the basket.
12	Flex the wrist at the end of the movement.
13	Make the ball spin in the opposite way.
14	No errors.

### PROCEDURES AND OUTLINE

After agreeing to participate, each participant was required to read and sign an Informed Consent before proceeding to the application of the MMSE.

The participants were divided in three experimental groups with gender counterbalancing control. *Group* ***G100*** received KP on 100% of their attempts; *Group* ***G66*** received KP on 66% of their attempts (having two attempts with KP and one attempt without KP); and *Group* ***G33*** received KP on 33% of their attempts (having two attempts without KP and one with KP). The provision of KP was based on the key elements for carrying out the throw from the checklist. Participants received information about the most important element of the movement to be corrected on the next attempt, which was named the *Critical Error.* This information was organized in order of priority (from 1 to 14, were 1 being the worst error and 14 is the correct movement) and the participants received the information according to their experimental group.

The acquisition phase was organized into three practice sessions held on alternate days with 30 trials on each day, with an interval of one minute after every 10 attempts, or 90 trials in total. Five minutes after the end of the third session, a retention test was immediately performed (R5), and 24 h after the third session a delayed retention test (R24) and a transfer test (T) were administered. Each participant performed 10 trials without KP in all tests (R5, R24, and T). Also, at the end of the third session, the participants were questioned about the relevance and use of the information given.

Before each session, each individual watched a videotape showing an experienced model demonstrating the execution of the throw, and he or she later received instructions on how to carry out the task. Next, each participant took five throws to familiarize themselves with the task.

In order to control the coherence of the Critical Error given to the participants by the experimenter during the learning process, twelve participants were randomly selected and, after watching a video of their first and last attempts at each practice session, the experimenter gave a KP for each attempt, which was compared with the KP assigned during the experimental phase. The Spearman test showed a correlation of *r* = 0.904 (*p* = 0.000) between the observations.

### STATISTICAL ANALYSIS

The statistical treatment was performed using *SPSS Statistics 17.0* software. The quantitative measure was analyzed using parametric tests (two way ANOVA). *Post hoc* comparisons were made with Tukey test with Bonferroni correction. The qualitative measures were analyzed by employing non-parametric tests, with Friedman’s ANOVA and, as a *post hoc* test, Wilcoxon’s test with Bonferroni’s procedure. For the intergroup comparisons the Kruskal–Wallis test was used with the Mann–Whitney *U* test as the *post hoc* test.

## RESULTS

**FIGURE 1:**
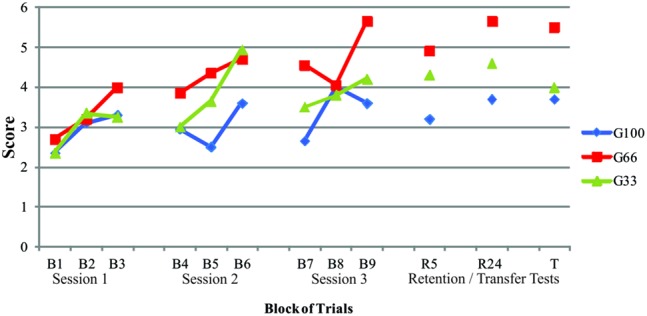
**Average performance of the sum of points (score) obtained during the acquisition phase (B1–B9), retention tests (R5 and R24), and transfer test (T) of G100, G66, and G33 groups**.

The quantitative measure was obtained from the sum of the scores achieved by the participants for each block of 10 attempts, and the mean of each block was taken for descriptive analysis. In order to see if there was change in mean scores over the time, during the acquisition phase, from B1–B9, results were analyzed with Repeated Measures ANOVA (9 blocks × 3 groups), so that we could infer that there was a performance improvement during practice. Another Repeated Measures ANOVA (4 blocks × 3 groups), from B9, R5, R24, to T, was performed to identify mean score change, although there was a 24 h gap between B9, R5, and R24, T; even in a similar task but with different distance, as Transfer task was. **Figure 1** illustrates the performance of the G100, G66, and G33 groups.

ANOVA showed a significant difference in the blocks (*F* = 9.679; *p* = 0.000), which was localized by Tukey’s multiple comparison analyses with Bonferroni’s correction. B1 was identified as being different from the remaining blocks (with the exception of B2); and B9 was different from the remaining blocks (except B6 and B8), adopting a significance level of *p* ≤ 0.05.

Intergroup analysis revealed a significant difference between the groups (*F* = 5.298; *p* = 0.008). The Tukey test confirmed that G66 was superior to the other groups (*p* = 0.006), indicating that this group achieved a higher number of non-zero scores.

With regard to the retention and transfer tests, there were no significant differences between the blocks (*F* = 1.270; *p* = 0.294), indicating that the groups maintained the same level of performance after an interval of 5 min (R5) and 24 h (R24), and even when the task was modified (T). In addition, a significant difference was found between the groups (*F* = 14.727; *p* = 0.001). Tukey’s test showed that G66 presented a better performance in the retention and transfer tests than G100 and G33.

**FIGURE 2:**
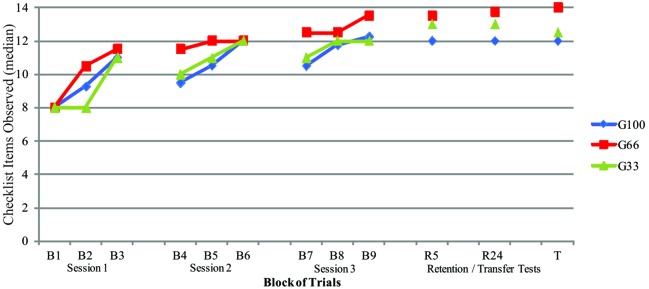
**Median of the checklist items observed during the acquisition phase (B1–B9), retention tests (R5 and R24), and transfer test (T) of G100, G66, and G33 groups**.

**Figure 2** illustrates the qualitative measure, or the checklist items observed (Critical Errors), which were analyzed by adopting the median as the central measure. Therefore a non-parametric analysis was taken. Friedman ANOVA was performed for each group from B1–B9, and later from B9, R5, R24, to T.

Friedman’s ANOVA found significant differences among the blocks in all groups (G100: *X*^2^ = 108.932, *p* = 0.000; G66: *X*^2^ = 98.048, *p* = 0.000; G33: *X*^2^ = 109.726, *p* = 0.000), that were localized by Wilcoxon’s test with Bonferroni’s procedure into the following blocks, respectively: in G100, B1 was different from B9, and both differed from all the other blocks; in G66, B1, and B2 were different from the remaining blocks and B9 was different from all blocks; in G33, B1 was different from all blocks, and B9 was different from all other blocks except B6 and B8 (*p* ≤ 0.005).

Friedman’s ANOVA with Wilcoxon’s *post hoc* test and Bonferroni’s procedure on blocks B9, R5, R24, and T, in each group, found that, in G100, there was a significant difference (*X*^2^ = 2.792; *p* = 0.040) between blocks B9 and T (*p* = 0.048). In G66, there was a significant difference (*X*^2^ = 14.044; *p* = 0.001) between blocks B9 and R24 (*p* = 0.008) and B9 and T (*p* = 0.018), while in G33 there was a significant difference (*X*^2^ = 6.906; *p* = 0.032), which was localized between blocks B9 and R24 (*p* = 0.022).

Intergroup comparisons carried out by the Kruskal–Wallis test showed that there was a significant difference [*X*^2^ (2.20) = 8.62; *p* = 0.013] between the groups on blocks B9, R5, R24, and T (*p* ≤ 0.004). The Mann–Whitney *U* test showed differences between groups G100 and G66 on blocks B9, R5, R24, and T (*p* ≤ 0.012), differences between groups G100 and G33 on block R24 (*p* ≤ 0.023) and between groups G66 and G33 a significant difference between blocks B9, R5, R24, and T (*p* ≤ 0.004).

Therefore, the inferential analysis showed that the participants in G66 presented a movement pattern qualitatively superior to those in the other two groups, especially in the last four blocks (B9, R5, R24, and T).

**FIGURE 3:**
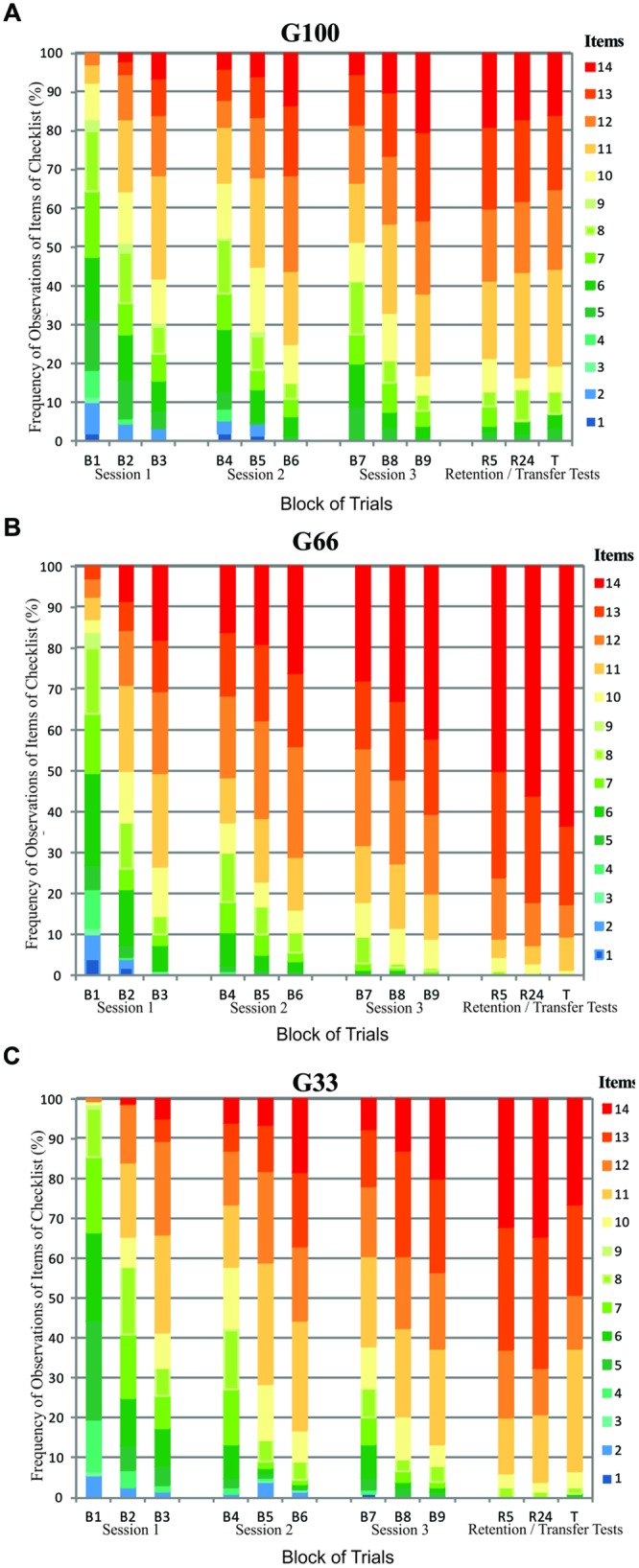
**Frequency of observations of the checklist items (%) during the acquisition phase (B1–B9), retention tests (R5 and R24), and transfer test (T) of G100 **(A)**, G66 **(B)**, and G33 **(C)** groups**.

In order to contrast the frequency of Critical Errors (or checklist items) in each group, we calculated the percentage of the times that each item of the checklist was observed for each group over each block of practice. This is because, the checklist items observed reflects individual’s movement pattern error, and we can see a qualitative change throughout practice, as an indicative of movement quality improvement. This is shown in **Figure 3**.

In general, participants in G66 presented a larger percentage of item 14, corresponding to the correct movement; in other words, they showed a movement pattern that was qualitatively superior to the participants in groups G100 and G33.

Spearman’s correlation showed that there was a positive and significant association between the movement pattern and the achievement of the task goal, which in this case was throwing the ball into the basket (G100: *r* = 0.677, *p* = 0.016; G66: *r* = 0.890, *p* < 0.00; and G33: *r* = 0.880, *p* < 0.001). Perhaps it should be pointed out that the participants in G100, who received KP on all attempts, presented a weaker association than those in G66 and G33.

## DISCUSSION

The objective of this work was to investigate the effect of the frequency of the provision of KP (33, 66, and 100%) on the acquisition of a motor skill in older individuals. One of the reasons for this work is that, according to Magill and Grodesky (2005) and Spirduso et al. (2005), strategies should be employed to facilitate learning among the older population. One example of such a strategy would be the provision of extrinsic feedback that offers information regarding the pattern of the movement, or what is known as KP. Although this reasoning is coherent, studies that investigate how these strategies help the older persons during learning are scarce.

In order to address this problem, several questions were initially raised that took into account some of the characteristics of the aging process. This was necessary because a high frequency of KP could benefit older individuals, since, according to some authors (Shephard, 1997; Magill and Grodesky, 2005) aging brings a certain decline in the capacity to recall information given, for example, by an instructor. On the other hand, we also asked whether perhaps high frequencies of KP might overload the information processing system of the older individuals, since, according to Magill and Grodesky (2005), during the teaching–learning process of a motor skill in older individuals, the amount of information given should not exceed their capacity to understand and retain the information provided.

However, the literature does not contain sufficient information to answer these concerns, since studies that adopted KP as a variable did not include older persons in their sample. Studies that manipulated KR with older individuals investigated the relative frequency of KR provision, but failed to reach a consensus over the ideal frequency for the provision of extrinsic feedback to the older persons (Behrman et al., 1992; Carnahan et al., 1996; Wishart and Lee, 1997; Gehring, 2008).

As discussed earlier, KR refers to information about the result of an action in relation to the intended aim, while KP refers to information about the movement pattern performed. In this sense, although dealing with distinct variables, KP and KR are both classified as belonging to the same category of feedback – extrinsic feedback – which consists of information originating from a source external to the body. In this way, a number of observations can be made regarding the results obtained in the present study and previous research on the frequency of KR provided to the older persons, since, according to Schmidt and Wrisberg (2004), the mechanisms of information processing for KP and KR appear to be the same.

From the results of Carnahan et al. (1996), it can be seen that the reduced frequency of 20% produced outcomes superior to those obtained with 100% feedback provision (KR). In the present work, this was not observed, since the group that received 100% KP provision (G100) was not significantly different from the group that received 33% KP (G33), in any phase of the experiment.

By contrast, in a study carried out by Wishart and Lee (1997), no significant differences were found between the results for different frequencies of KR provision (100 and 67%), while in the present study it was observed that the group that received 66% KP (G66) gave superior outcomes to the other groups (G100 and G33) on both the qualitative and the quantitative measures during the retention and transfer tests. The superiority of the frequency of 66% is the most notable result of this study.

Studies by Salthouse (1979, 1994, 2000) and Gunning-Dixon et al. (2009) suggest that there are changes in the information processing capacity throughout life. Such changes would interfere directly in the capacity of older persons to cope with information, such as KP, coming from an external source.

When our results are considered, the analysis of the evolution of Critical Errors made by each group as a result of the provision of KP (**Figure 3**) suggests a noticeable effect of practice. This is because the movement pattern became qualitatively better as the participants were allowed to practice the basket free throw. This clearly shows that the unpolished errors in the checklist were progressively reduced, until the provision of KP led to the correct movement (item 14).

In addition, a positive association was also detected between the movement pattern and the performance in all experimental groups. This means that it was possible to establish a relationship between the movement pattern and the task goal. In other words, as the movement pattern qualitatively improved, the score increased. Group G66 obtained the highest correlation and, also, presented the highest number of people reaching item 14, which corresponds to the movement pattern which is considered to be correct. This frequency of KP provision enabled this group to be more consistent with regard to the movement pattern when compared to the other groups. In contrast, the provision of KP provision on all attempts led group G100 to achieve a more unstable performance with greater variability in the movement pattern.

One aspect that deserves to be highlighted is that, during the interval between practice sessions (that is, after a period without practicing the task), groups G33 and G100 appeared to suffer a marked deterioration in performance. At least one block of 10 attempts was necessary to recover their performance and the movement pattern presented in the previous session. However, the participants in G66 seemed to be less affected by this interval, since when returning from the interval between practice sessions they presented a similar performance to that in the last block of the previous session. Thus this shows that the provision of KP on 66% of attempts supported the process of consolidation.

Practice is essential for the acquisition of motor skills; however, even after the end of practice the brain continues to process information. When the entire process of motor acquisition is taken into account, two aspects should be considered: the learning process itself (acquisition plus retention); and the capacity to transfer what is learned to new conditions and variants of the task (transfer). The present study tested the capacity of the older persons to transfer the learned skill, and their capacity to adapt to a disruption.

Despite the age-related decline in the acquisition of motor skills, the results indicate that the older persons are capable of adapting to a new demand by using their prior experience. This result goes against statements made by Seidler et al. (2006), who suggested that the subjacent processes that contribute to motor acquisition and transfer are distinct and, moreover, that these processes are affected by age in distinct ways. This dissociation was also documented by Seidler (2006, 2007).

If we take the explanatory hypotheses formulated to explain the KR data (Henry, 1968; Salmoni et al., 1984; Winstein and Schmidt, 1990), our results do not support the hypothesis of similarity or specificity, because the group G33 practiced under conditions closer to those of the test, in the sense they had less feedback information than the other groups, but did not have superior performance. On the other hand, the explanatory hypotheses predict that frequent feedback may lead the learner to excessive instability during practice, because it causes frequent adaptations or modifications to the performance, and consequently, make it difficult to develop the capacity for stability on retention and on transfer. If this is true, G100 should be inferior to G33, but inferential analysis showed that G100, in which the participants received KP on all their attempts, obtained similar results to G33, which was the group with the lowest frequency of KP.

The guidance hypothesis is based on the idea that if the learner receives KR on each attempt then this leads to dependency on the information; inhibiting other relevant processing activities in the process of motor learning, inhibiting the capacity to detect and correct errors, and causing the blocking of memory recall which would threaten the development of an action plan. This means that the hypothesis predicts that G100 should be inferior to both other groups, G66 and G33, but again this was not the case. This is because the members of G100 did not become dependent on the KP information as would be expected under the guidance hypothesis. They were capable of maintaining their performance, as seen in the last block of the acquisition phase and the tests of retention and transfer, showing themselves to be autonomous in relation to extrinsic feedback. One possible explanation for the inferior performance of G33 is related to the role of sources of intrinsic information, which originate from the sensory system.

It is relevant to emphasize that receiving extrinsic feedback does not guarantee its effective use, because information is only transmitted when the transmission will mean that uncertainty is reduced. Moreover, the fact that data is available does not guarantee that it will be interpreted by the individual. If the data are not properly interpreted, they are characterized as a message and not as information. For this reason, the learner must be able to use the information to reduce uncertainties and, also, must want to use it (Meira, 2005).

It seems that the participants in G33 did not receive enough extrinsic feedback (to add to their intrinsic information) to generate effective responses. By contrast, those in G100 were not able to deal with the information received in every trial, which maybe caused an overloaded system. Therefore, the provision of KP on 66% of attempts caused the information to be added to the individual’s sensory information and, perhaps, this helped the perception of intrinsic feedback, while at the same time, removing a large amount of uncertainty. This regimen for the provision of KP may also have helped the older persons to remember critical movements in the execution of the basket free throw without overloading their memory, besides allowing them some time (in the attempts without extrinsic feedback) to consolidate this information. This would enable the formation of mechanisms of error detection and correction that would be efficient and relevant for when the KP was completely removed in the tests of retention and transfer.

One could argue that the explanatory hypothesis discussed earlier, were established on KR studies, while the present study is about the KP effect on learning. The reason for these is that it seems that this is a pioneer study on the KP effect related to older population, and it seemed applicable to bring such information.

In this sense, as none of the explanatory hypotheses discussed here offer support for the results found in this study, perhaps it could be said that there is a need for another type of argument to explain how older individuals cope with extrinsic feedback during the learning process. It is possible that such an explanatory hypothesis might be characterized as an optimal processing hypothesis. Finally, it is necessary to examine this question through other reasoning, exploring the provision of extrinsic feedback provided with intermediate frequencies of KP, or even the provision of other forms of feedback, such as self-controlled feedback.

In conclusion the groups that received KP on 33 and 100% of their attempts performed similarly throughout the learning process of the basketball free throw. The results cannot be explained by the explanatory hypotheses of instability and guidance, and older individuals appear to require a particular frequency of KP provision when learning a motor skill.

## Conflict of Interest Statement

The authors declare that the research was conducted in the absence of any commercial or financial relationships that could be construed as a potential conflict of interest.
